# Lateral insertion is a good prognostic factor after in situ fixation in slipped capital femoral epiphysis

**DOI:** 10.1186/1471-2474-15-317

**Published:** 2014-09-26

**Authors:** Shigeo Hagiwara, Junichi Nakamura, Makoto Kamegaya, Takashi Saisu, Jun Kakizaki, Seiji Ohtori, Shunji Kishida, Kazuhisa Takahashi

**Affiliations:** Department of Orthopaedic Surgery, Graduate School of Medicine, Chiba University, 1-8-1 Inohana, Chuo-ku, Chiba City, Chiba 260-8677 Japan; Chiba Child and Adult Orthopaedic Clinic, 3-24-2 Oyuminominami, Midori-ku, Chiba City, Chiba 66-0007 Japan; Division of Orthopaedic Surgery, Chiba Children’s Hospital, 579-1 Heta-chou, Midori-ku, Chiba City, Chiba 266-0007 Japan

## Abstract

**Background:**

In situ fixation (ISF) is standard treatment for slipped capital femoral epiphysis (SCFE) to stabilize the epiphysis and to prevent further slip. The aim of this study was to clarify the incidence of slip progression after ISF and its prognostic factors.

**Methods:**

We retrospectively reviewed 53 hips in 49 consecutive SCFE patients who underwent single screw ISF and were followed until physeal closure. Clinical and radiographic findings were viewed to assess progression of the posterior tilting angle (PTA).

**Results:**

Mean PTA was 33.4 degrees (range, 18 to 75 degrees) at ISF and 35.9 degrees (range, 18 to 75 degrees) at physeal closure with progression of PTA of 2.5 degrees (range, -2 to 19 degrees). Slip progression occurred in 28 of 53 hips (53%), and more than five degrees of progression occurred in 14 hips (26%). Multiple regression analysis revealed that point of screw insertion (one point for lateral and two points for medial) was a significant prognostic factor for progression of the slip by the following formula: (progression of PTA) = -1.523 + 2.701 × (point of screw insertion), R^2^ = 0.148, *p* = 0.005.

**Conclusions:**

The current study showed that a screw inserted from the lateral side to the intertrochanteric line prevented postoperative slip progression.

**Electronic supplementary material:**

The online version of this article (doi:10.1186/1471-2474-15-317) contains supplementary material, which is available to authorized users.

## Background

Slipped capital femoral epiphysis (SCFE) is the most frequent hip problem in adolescents [1–4]. Mechanical stress from body weight is thought to be responsible for progression of posterior slip of the epiphysis, leading to groin pain and external rotation of the lower extremity [5]. Morphology of the proximal femur has been shown to associate with the femoro-acetabular impingement in adolescents and young adults [6–8], resulting in osteoarthritis of the hip [9–11]. Therefore, early diagnosis and optimal treatment is crucial to improve the outcome.

Sometimes osteotomy is needed to realign the proximal femur [12]. In situ fixation (ISF) of SCFE is widely accepted as an initial treatment to stabilize the capital epiphysis and to prevent further slip [13–15]. However, slip progression can occur even after ISF and several osteotomy procedures for realignment of the proximal femur have been proposed [12]. The aim of this study was to clarify the incidence of slip progression after ISF, and to identify the prognostic factors that can predict slip in SCFE patients.

## Methods

The study protocol was approved by the institutional review board (Research Ethics Committees, Chiba Children and Adult Orthopaedic Clinic) and all the patients gave written informed consent. This retrospective observational cohort study included only SCFE patients who underwent single screw ISF as the initial treatment from 1996 to 2010 and were followed until physeal closure. Patients with avascular necrosis, endocrine disorders, multiple screw fixation, and preventive fixation for asymptomatic contralateral hip were excluded. Joint- based analysis was applied in this study. The patients were admitted to the hospital and kept in bed with one kg of positional traction until the operation.

The Lauenstein method was applied for the lateral image as follows: care was always taken to keep the patient’s trunk in a semilateral decubitus position, 45 degrees leaning to the affected side and keeping the patient’s thigh on the platform with 90 degrees hip flexion. posterior tilting angle (PTA) was measured by neck-shaft angle using the Southwick procedure [16]. ISF was basically indicated when PTA was 40 degrees or less. In cases of PTA more than 40 degrees, ISF could be indicated only if the femoral head was round on the anterior-posterior X-ray image and the range of motion was well maintained over 90 degrees of flexion. Each patient was carefully laid on the operating table without traction under general anesthesia. Attention was paid throughout the operation not to reduce the slipped femoral epiphysis by traction or internal rotation and not to penetrate a guide wire into the hip joint space without exiting the femoral neck and re-entering the femoral head, damaging the posterior vascular structures, as viewed under the image intensifier. Then, a Richard titan cannulated screw with 16 mm thread (6 threads) and 6.5 mm diameter (Smith&nephew, Memphis) was inserted. ISF was performed by multiple surgeons under a single supervisor. Postoperatively, patients were restricted to non-weight bearing for three to six weeks using a wheel chair or crutches depending on the stability. Then, full weight bearing was permitted at six weeks in chronic SCFE, and at three months in both acute and acute on chronic types of SCFE. PTA was continuously measured from before ISF to physical closure.

### Statistical analysis

**Figure 1:**
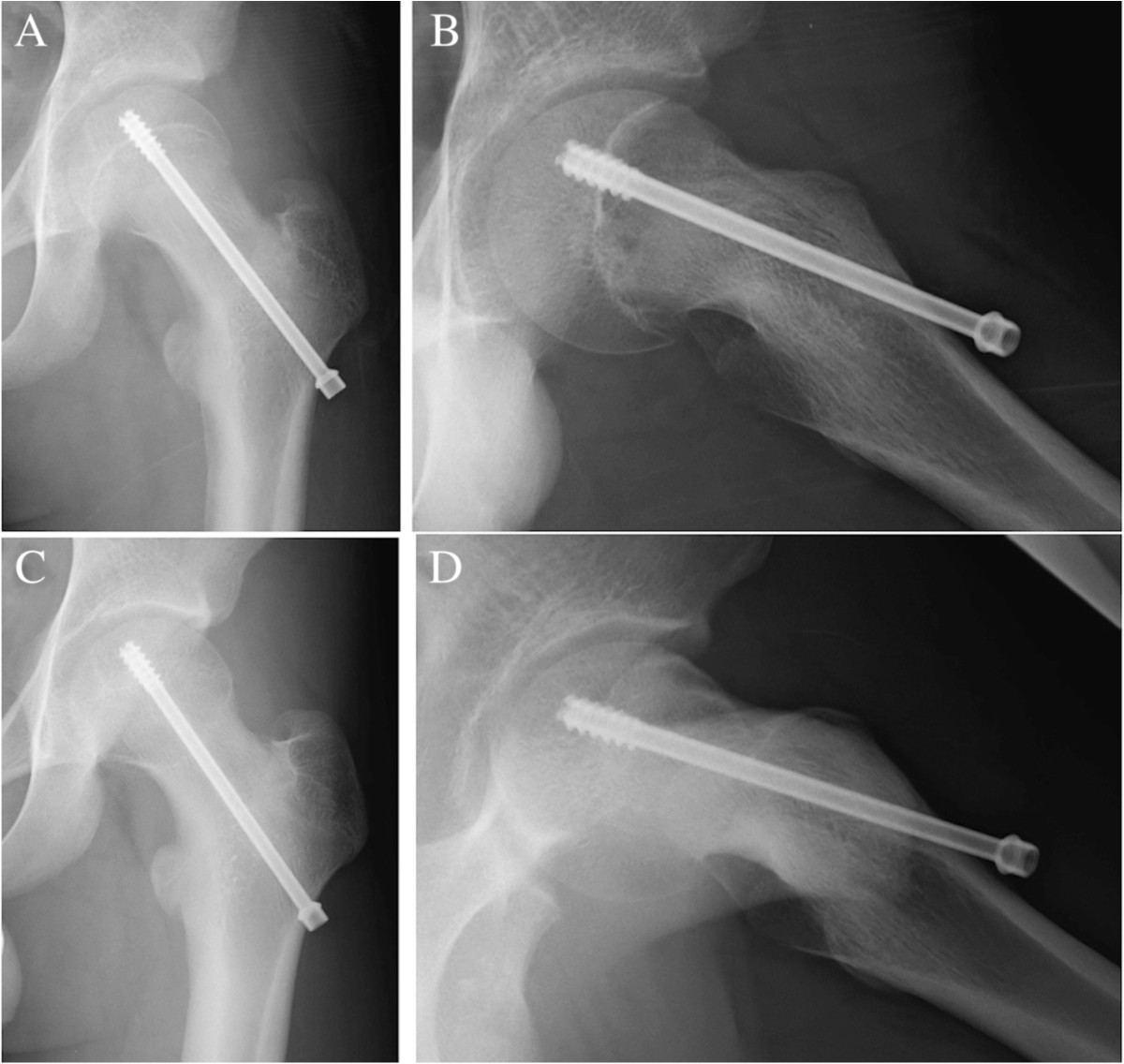
**Radiographs of the left hip in a 13-year-old boy with SCFE.** Anteroposterior **(A)** and lateral **(B)** radiographs immediately after in-situ fixation (ISF) for acute on chronic and stable type hips at 42 degrees of posterior tilting angle (PTA). A single screw was inserted from the lateral point. Anteroposterior **(C)** and lateral **(D)** radiographs at physeal closure. PTA was maintained without progression of the slip.

**Figure 2:**
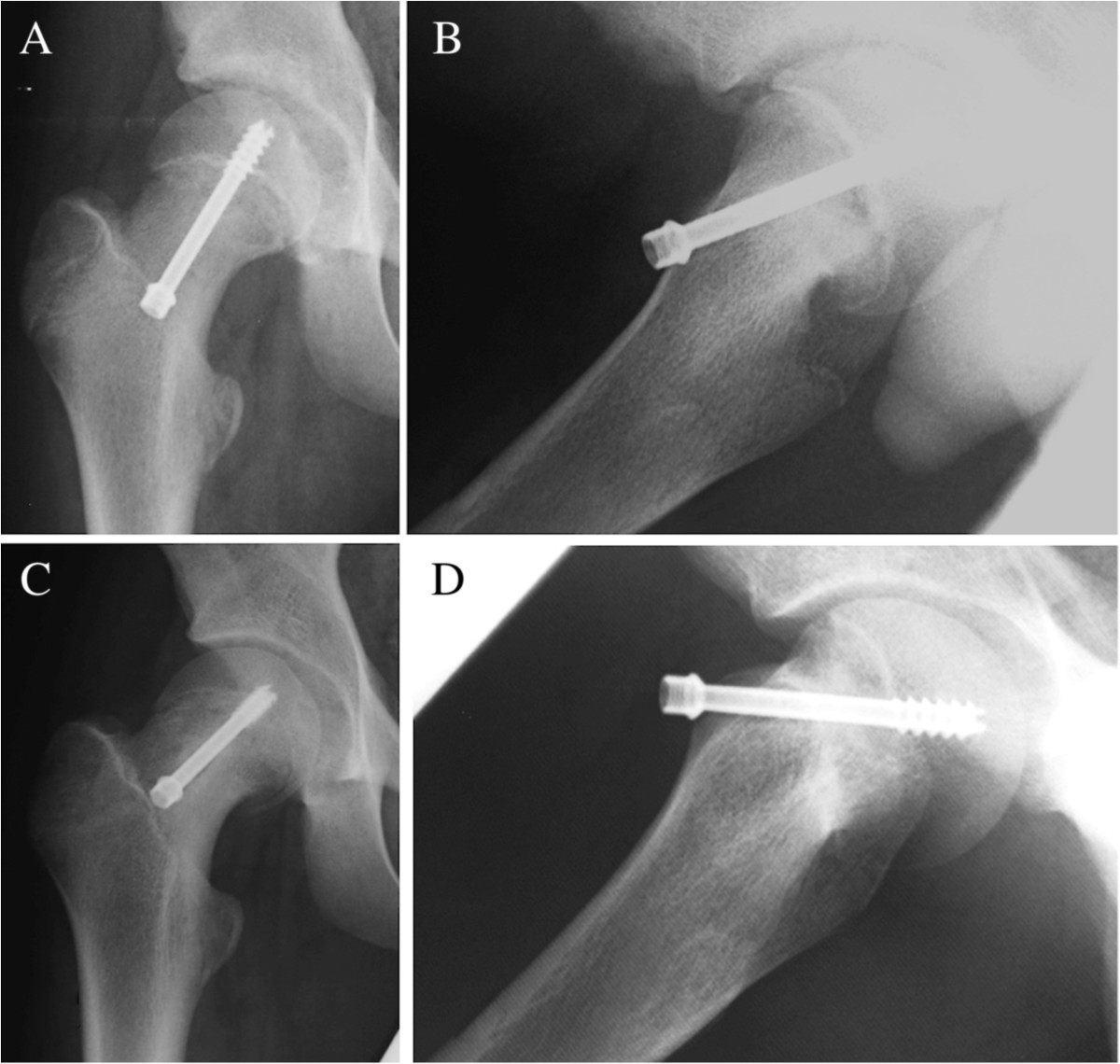
**Radiographs of the right hip in a 14-year-old boy with SCFE.** Anteroposterior **(A)** and lateral **(B)** radiographs immediately after ISF for chronic and stable type hips at 38 degrees of PTA. A single screw was inserted from the medial point to the center of the epiphysis perpendicularly. Anteroposterior **(C)** and lateral **(D)** radiographs at physeal closure. Slippage of the epiphysis had progressed to 43 degrees of PTA 17 months after surgery.

**Figure 3:**
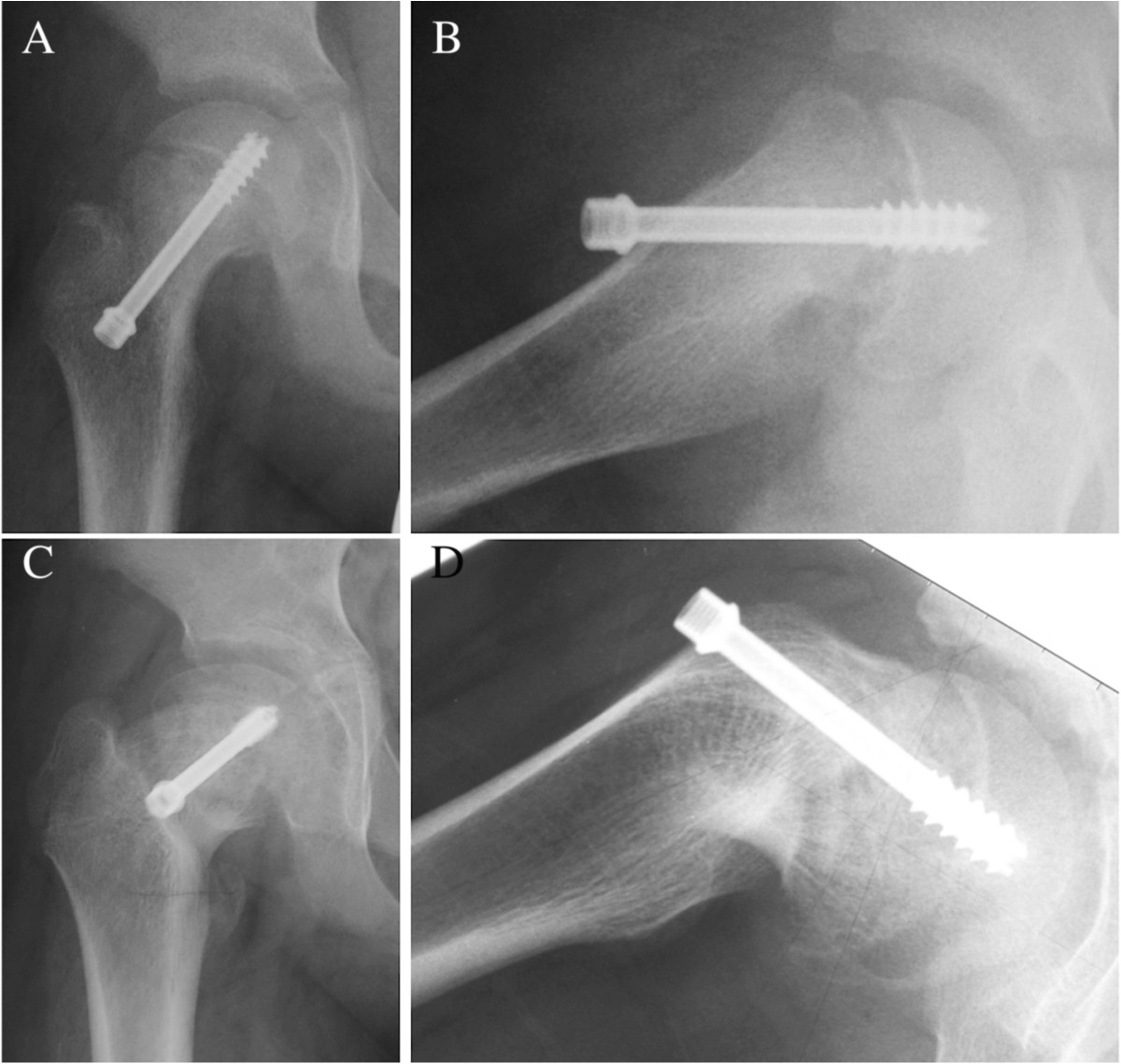
**Radiographs of the right hip in a 10-year-old boy with SCFE.** Anteroposterior **(A)** and lateral **(B)** radiographs immediately after ISF for chronic and stable type hips at 37 degrees of PTA. A single screw was inserted from the medial point to the center of the epiphysis perpendicularly. Anteroposterior **(C)** and lateral **(D)** radiographs at physeal closure. PTA had progressed to 56 degrees 18 months after surgery due to excessive retroversion of the femoral neck.

Radiological evaluation of PTA included the screw position [16, 17], the distribution of threads across the epiphysis [18], and the point of screw insertion [19]. Slip progression was defined as a change of more than five degrees of PTA based on Rao et al. [20]. The screw position was determined using the Aronson and Carlson system [17]: 1 point, the central axis of the screw is located over the center line of the femoral head or within a distance equal to one-half the diameter of the screw; 2 points, the distance between the axis of the screw and center line of the femoral head is between one-half and one screw-diameter; and 3 points, the axis of the screw is located more than one screw-diameter from the center line of the femoral head. The distribution of threads across the epiphysis was determined using the method of Upasani [18]: 1 point, 40-60% of the threads engage the physis; 2 points, < 40% or > 60% of the threads engage the physis. The point of screw insertion was defined as follows: 1 point, lateral to the intertrochanteric line both in anteroposterior and lateral views (Figure 1); and 2 points, medial to the intertrochanteric line (Figures 2 and 3). Goodwin’s classification [19] of screw head positioning was modified by the exact screw insertion. The radiographic evaluations were independently performed by two authors; however, the first author’s decision was adopted regarding other parameters. Cohen’s quadratic weighted kappa statistic [21] was used to assess the inter-observer and intra-observer reliabilities of the evaluation regarding the screw position, the point of screw insertion, and the distribution of threads. Intra-class correlation coefficients (ICC) were used to assess the inter-observer and intra-observer reliabilities of the evaluation of PTA. Step-wise multiple regression analysis was performed to determine the prognostic factors for slip progression after ISF. Candidate factors were the age at ISF, body mass index (BMI), onset pattern (1 point in chronic, 2 points in acute on chronic, and 3 points in acute), grade of stability (1 point in stable and 2 points in unstable), PTA at ISF, the screw position, distribution of threads across the epiphysis, and the point of screw insertion. A probability value <0.05 was considered to be significant with SPSS 16.0 J (SPSS Inc., Chicago, IL).

## Results

We registered 112 consecutive hips in 98 SCFE patients (75 boys and 23 girls) from 1996 to 2010. Initially, ISF was performed on 77 hips in 64 SCFE patients. Subsequently, we excluded four hips in four patients with avascular necrosis, six hips in three patients with endocrine disorders, six hips in six patients with double screw fixation, and six hips with preventive fixation for asymptomatic contralateral hip. Of the remaining 55 hips, 53 hips (24 right, 21 left, and 4 bilateral) in 49 patients (40 boys and 9 girls) were observed until physeal closure with a 96% follow-up rate. The mean time to physeal closure, i.e. follow-up period, was 12.9 months (range, 3 to 30 months).

Mean age at ISF was 11.8 years (range, 8 to 14 years) and mean BMI was 24.7 (range, 14.6 to 36.2). Mean duration from first visit to our hospital to ISF was 9.5 days (range, 1 to 52 days). Onset of symptoms was acute in 12 hips, acute on chronic in 12, and chronic in 29. Functionally, 48 hips were stable and 5 were unstable.

Cohen’s quadratic weighted kappa for inter-observer reliability was 0.700 for the screw position, 0.886 for the point of screw insertion, and 0.798 for the distribution of threads. ICC was 0.977 in PTA. Cohen’s quadratic weighted kappa for intra-observer reliability was 0.938 for the screw position, 0.876 for the point of screw insertion, and 0.588 for the distribution of threads. ICC was 0.989 in PTA.

Mean PTA at ISF was 33.4 degrees (range, 18 to 75 degrees). Mean PTA at physeal closure was 35.9 degrees (range, 18 to 75 degrees) with progression of PTA of 2.5 degrees (range, -2 to 19 degrees). We performed an additional osteotomy after physeal closure in four hips in which PTA was more than 45 degrees and range of motion was restricted with the Drehmann sign [12]. There were no screw penetrations into the joint space and or fractures around the screws. Screw position was center in 29 hips, next to center in 19 hips, and apart from center in five hips. The distribution of threads across the epiphysis was between 40 and 60% in 33 of 53 hips. The point of insertion was lateral in 30 hips and medial in 23 hips.

**Figure 4:**
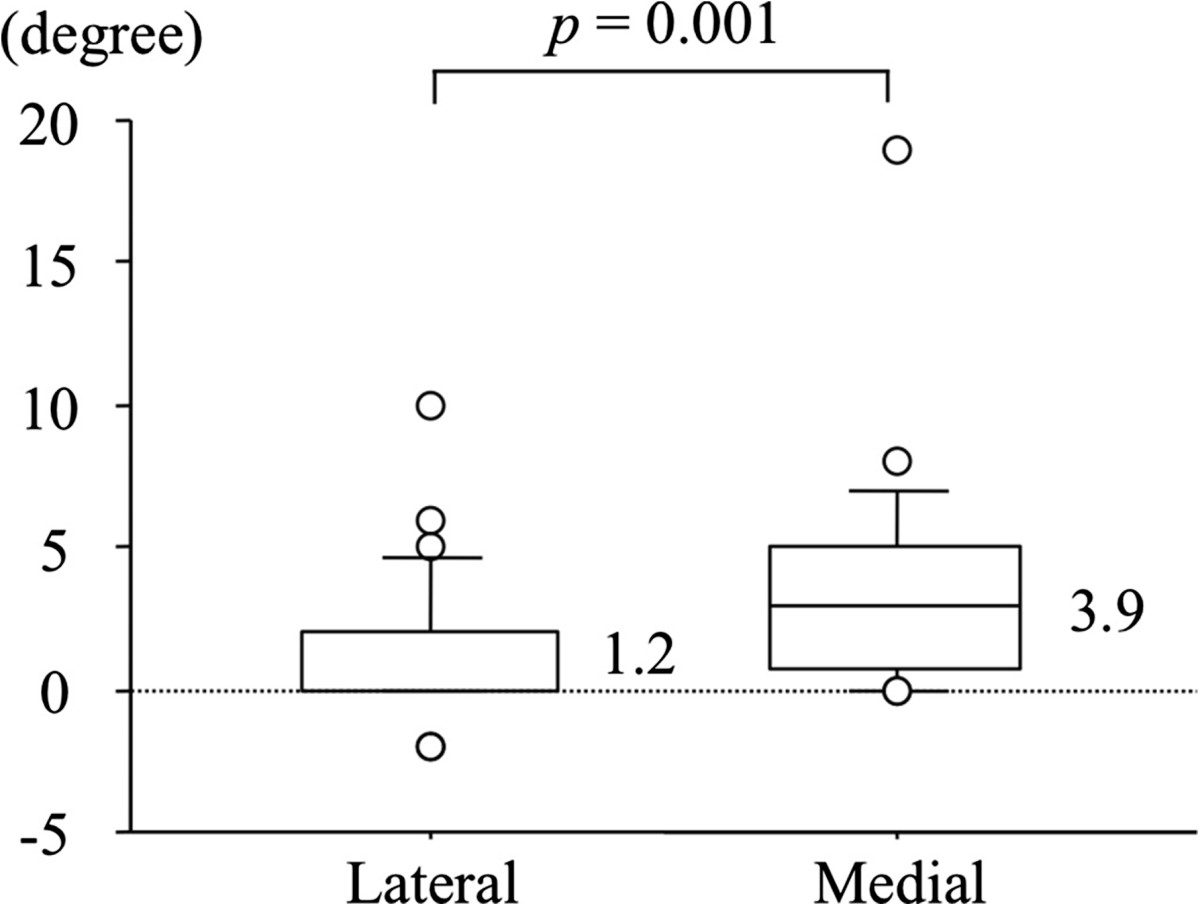
**Progression of PTA related to the point of screw insertion.** The point of screw insertion was judged by whether it was medial or lateral to the intertrochanteric line on anteroposterior and lateral radiographs. There was a statistically significant difference between the medial and the lateral point of screw insertion. Boxplots display the median, the upper and lower quartiles. Whisker lines from box plots indicate the percentile 10% and 90%. (Mann–Whitney *U* test, *p* = 0.001).

Slip progression occurred in 28 of 53 hips (53%), and more than five degrees of progression occurred in 14 hips (26%). Table 1 shows comparison of slip-retention group (five degrees or less) and slip-progression group (more than five degrees). Both PTA at ISF and at physeal closure were not significantly different between the slip-retention group and the slip-progression group. Progression of PTA was naturally significantly larger in the slip-progression group than in the slip-retention group (6.9 degrees versus 0.9 degrees, *p* = 0.001). Rate of lateral insertion was significantly higher in the slip-retention group than in the slip-progression group (*p* = 0.011). Acute on chronic type of SCFE was more in the slip-retention group than in the slip-progression group with a statistical difference (*p* = 0.043). Furthermore, Table 2 shows comparison of the lateral insertion group and the medial insertion group. PTA at ISF was significantly larger in the medial group than in the lateral group (37.7 degrees versus 29.6 degrees, *p* = 0.004). PTA at physeal closure was also significantly larger in the medial group than in the lateral group (41.6 degrees versus 30.8 degrees, *p* = 0.001). Consequently, progression of PTA was 3.9 degrees for the medial group and 1.2 degrees for the lateral group with a statistical difference (*p* = 0.001, Figure 4).

**Table 1:** **Comparison of slip-retention group and slip-progression group**

	Retention group (39 hips)	Progression group (14 hips)	*p* value
Age (years)	11.9	11.6	0.420*
BMI	24.2	26.1	0.118*
PTA at ISF (degrees)	33.7	32.6	0.944*
PTA at physeal closure (degrees)	34.6	39.5	0.077*
Progression of PTA (degrees)	0.9	6.9	0.001*
SCFE type (acute: acute on chronic: chronic)	9:12:18	3:0:11	0.043¶
Stability (stable: unstable)	35:4	13:1	1.000§
Screw position (1:2:3)†	22:14:3	7:5:2	0.759¶
Distribution of threads (1:2)‡	17:22	3:11	0.203§
Point of insertion (lateral:medial)	25:14	3:11	0.011§
Time for full-weight bearing (weeks)	4.8	3.4	0.165*
Time for physeal closure (months)	12.1	15.4	0.088*

Slip-retention group means five degrees or less of PTA increase and slip-progression group means more than five degrees. †1 point, the central axis of the screw is located over the center line of the femoral head or within a distance equal to one-half the diameter of the screw; 2 points, the distance between the axis of the screw and center line of the femoral head is between one-half and one screw-diameter; and 3 points, the axis of the screw is located more than one screw-diameter from the center line of the femoral head. ‡1 point, 40-60% of the threads engage the physis; 2 points, < 40% or > 60% of the threads engage the physis. *Mann-Whitney’s *U* test, ¶Pearson’s χ^2^ test, §Fisher’s exact probability test.

**Table 2:** **Comparison of lateral insertion group and medial insertion group**

	Lateral group (28 hips)	Medial group (25 hips)	*p* value
Age (years)	12.0	11.7	0.274*
BMI	23.9	25.6	0.113*
PTA at ISF (degrees)	29.6	37.7	0.004*
PTA at physeal closure (degrees)	30.8	41.6	0.001*
Progression of PTA (degrees)	1.2	3.9	0.001*
Ouctome (slip retention: slip progression)	25:3	14:11	0.011§
SCFE type (acute: acute on chronic: chronic)	8:9:11	4:3:18	0.053¶
Stability (stable: unstable)	25:3	23:2	1.000§
Screw position (1:2:3)†	19:6:3	10:13:2	0.067¶
Distribution of threads (1:2)‡	12:16	8:17	0.571§
Time for full-weight bearing (weeks)	4.0	4.9	0.378*
Time for physeal closure (months)	13.5	12.4	0.513*

†1 point, the central axis of the screw is located over the center line of the femoral head or within a distance equal to one-half the diameter of the screw; 2 points, the distance between the axis of the screw and center line of the femoral head is between one-half and one screw-diameter; and 3 points, the axis of the screw is located more than one screw-diameter from the center line of the femoral head. ‡1 point, 40-60% of the threads engage the physis; 2 points, < 40% or > 60% of the threads engage the physis.

*Mann-Whitney’s *U* test, ¶Pearson’s χ^2^ test, §Fisher’s exact probability test.

Multiple regression analysis revealed that point of screw insertion (one for lateral and two for medial) was an independent prognostic factors for slip progression, and could be predicted by the following formula: (progression of PTA) = (progression of PTA) = -1.523 + 2.701 × (point of screw insertion), R^2^ = 0.148, *p* = 0.005.

## Discussion

A lateral point of screw insertion was an independent prognostic factor for slip progression after ISF in SCFE patients. The current study showed that a single screw inserted from lateral to intertrochanteric line prevented postoperative slip progression without the risk of screw penetration into the joint. One of the reasons may be because the thickness of the cortical bone at the proximal femoral metaphysis increases from the medial to the lateral aspect and the screw can go through a longer distance in the metaphysis, resulting in secure stability of the screw (Figure 1). Goodwin et al. suggested that a screw inserted from medial to the intertrochanteric line perpendicular to the physis caused acetabular impingement on the anterior acetabular rim and the screw head in moderate to severe SCFE patients [19]. Goodwin’s classification based on screw head positioning [19] was modified in the current study to the exact screw insertion using anteroposterior and lateral radiographs. The screw head positioning was determined by the length of the screw as well as the exact screw insertion. Thus, we regarded the point of insertion itself as more reliable. Another advantage of lateral insertion is that it is easy to remove the screw at the time of additional osteotomy by a single incision in severe SCFE. We recommend that lateral insertion technique is effective for ISF in SCFE patients.

However, best practices for ISF have been controversial [22–25]. Several studies have advocated medial insertion of the screw to penetrate the physis perpendicular at the center of the epiphysis [17, 26, 27]. Aronson et al. recommended that the screw should be inserted from the anterior femoral neck perpendicular to the physis at the center of the femoral head through an anterolateral approach with the patient in the supine position to prevent screw penetration into the joint [17]. According to this method, the point of screw insertion is medial in severe SCFE. Riley et al. reported 26% of 308 SCFE hips showed screw-related complications and claimed that a lateral insertion of the screw could damage the posterior wall of the femoral neck and posterior superior retinacular vessels, which provide the major blood supply of the femoral head, and the screw could break when it exits the femoral neck before entering the femoral head [27]. In this study, PTA at ISF was expectedly greater in the medial group than in the lateral group with a statistical difference. However, we still hold a warning for the medial insertion because slip progression after ISF would likely happen. It is true that lateral insertion is technically demanding in severe SCFE, but using an image intensifier intra-operatively, the screw positioning can be easily confirmed and such penetration can be avoided [28]. Moreover, three-dimensional computed tomography is helpful for preoperative planning and navigation surgery [29].

Ward et al. reported that mean time to physeal closure was 13 months with single screw fixation and longer with eccentric placement of the screw [26]. In general, the longer time to physeal closure is a likely risk factor for further slippage, as the slip could progress during this period. In this study, time to physeal closure was consistent with Ward’s report, but there was no significant difference between the slip-retention group and the slip-progression group or between the lateral and the medial insertion groups. Sanders pointed out that when a screw was inserted into osteopenic bone in the proximal femoral metaphysis, it was likely to loosen and accelerate the slippage of the epiphysis [23] (Figures 2 and 3).

Some biomechanical and clinical studies suggest that double screws rather than a single screw is better, due to increased rotational stability under torsional loading conditions [22, 30, 31]. Other studies have recommended a single screw, and have shown excellent clinical results and a lower complication rate in ISF [17, 32]. Single screw fixation is technically easier and safer, and decreases the prevalence of penetration into the joint.

Upasani et al. reported that maximum stability was gained when 40%-60% of the threads engaged the epiphysis using a 16 mm-thread single screw [18]. Carney et al. recommended that five or more threads should engage the epiphysis [24]. In our study, the distribution of the threads was not a prognostic factor.

Patient characteristics such as the growth spurt, endocrinologic disorders, unstable slip, or acute-on-chronic slip were previously reported as risk factors, although they were not found to be significant factors in this study.

There were several limitations to this study. First, because of its retrospective nature, patient characteristics varied, and each operation was performed according to the surgeon’s preference and best judgment. A prospective comparative trial is desirable to validate our results. Second, all the measurements were based on simple radiographs, and may have included some measurement error. However, inter-observer and intra-observer measurements were shown statistically to be closely related. Thus, we conclude that radiographic evaluation is still the gold standard for pediatric patients. Further study is needed using computed tomography or magnetic resonance imaging to determine whether these are more reliable or more accurate.

## Conclusions

The incidence of slip progression after ISF in SCFE was 53% (28 of 53 hips). More than five degrees of progression occurred in 14 hips (26%). Lateral screw insertion was a favorable prognostic factor in ISF.

## Authors’ original submitted files for images

Below are the links to the authors’ original submitted files for images. Authors’ original file for figure 1 Authors’ original file for figure 2 Authors’ original file for figure 3 Authors’ original file for figure 4
