# Supplementary figures and images for: Lateral insertion is a good prognostic factor after in situ fixation in slipped capital femoral epiphysis
Source: BMC Musculoskelet Disord. 2014 Sep 26;15:317. doi: 10.1186/1471-2474-15-317 (PMC4189656; doi:10.1186/1471-2474-15-317)

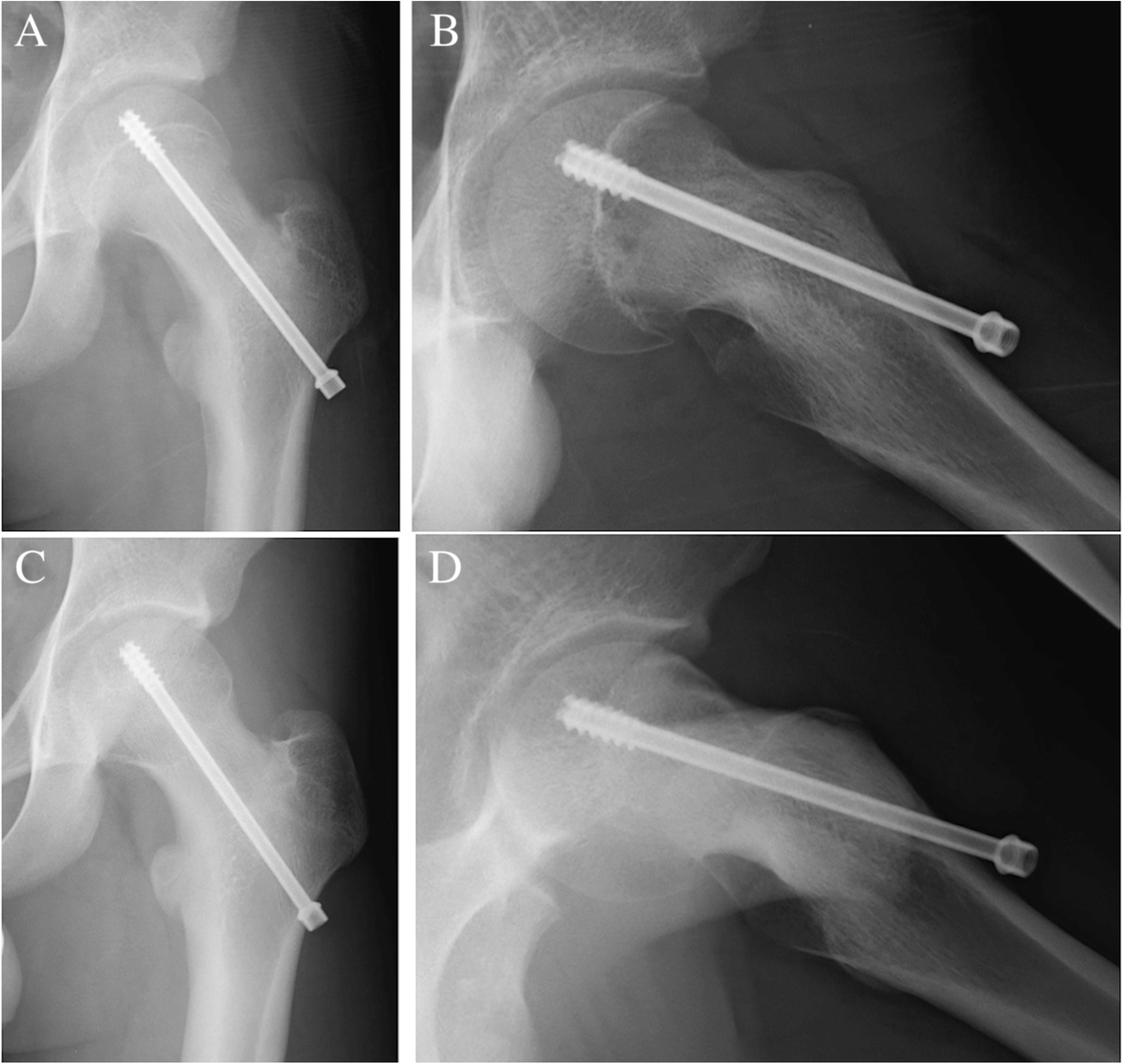

Supplement: Supplementary file 1 — Authors’ original file for figure 1 [file 12891_2014_2250_MOESM1_ESM.tif]

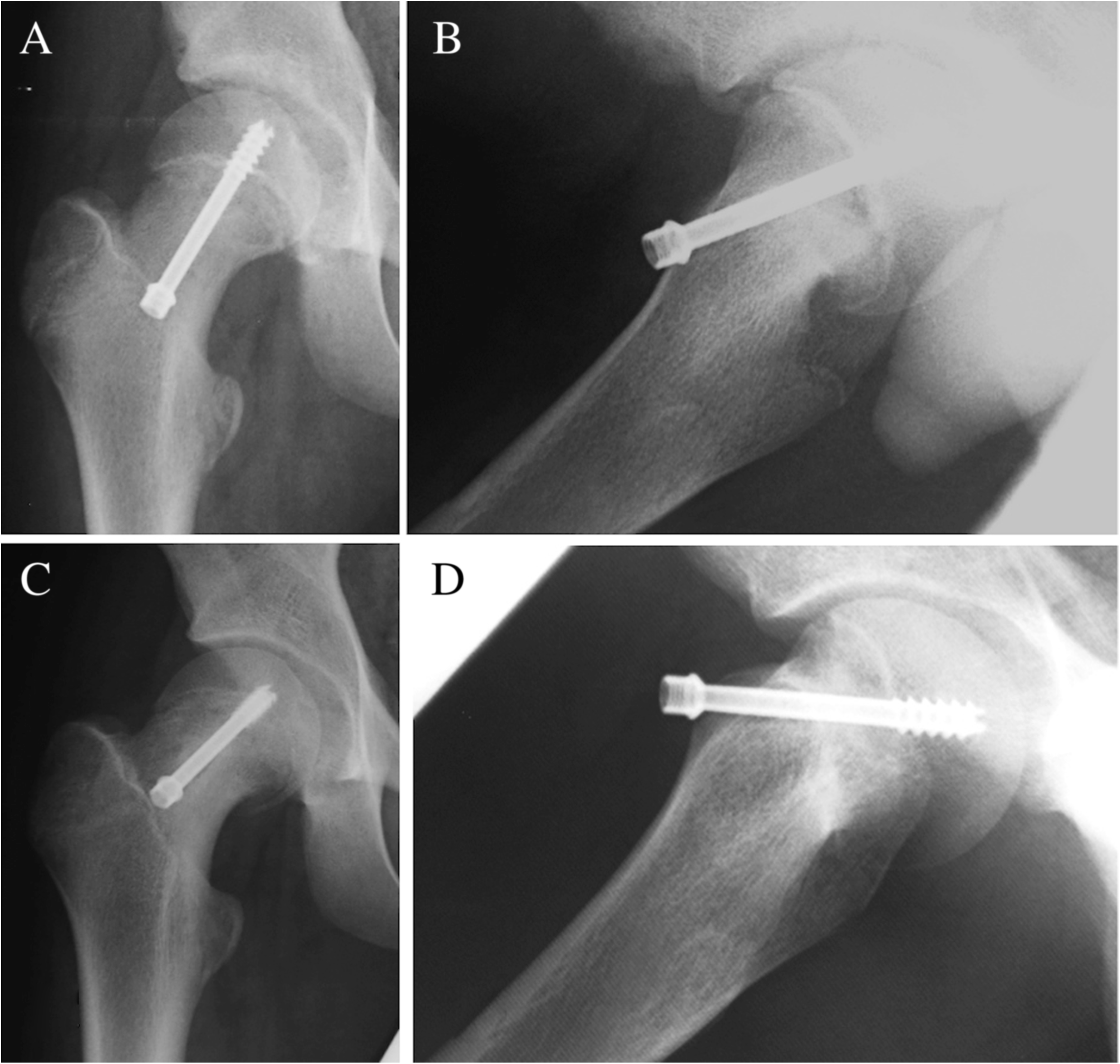

Supplement: Supplementary file 2 — Authors’ original file for figure 2 [file 12891_2014_2250_MOESM2_ESM.tif]

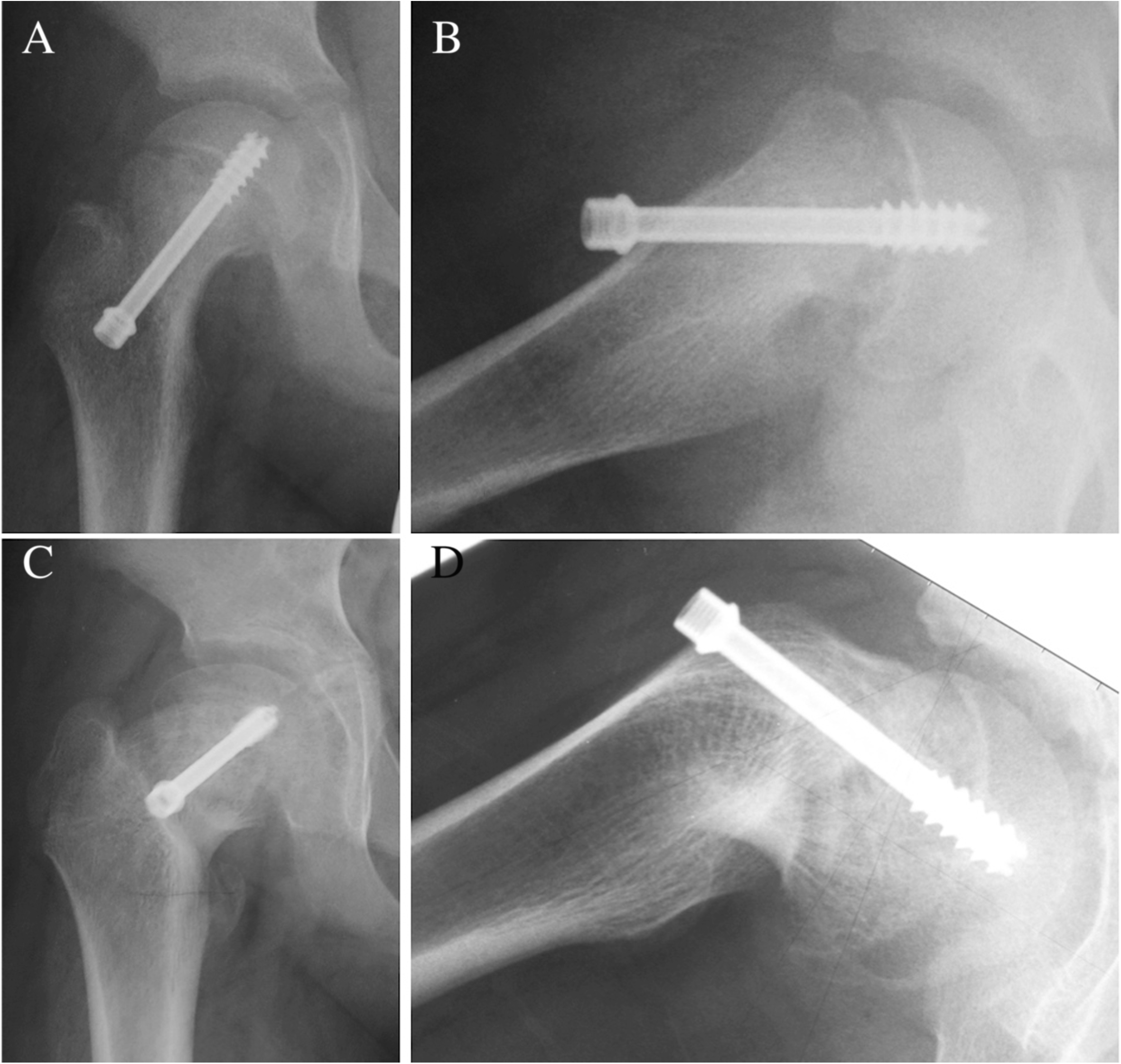

Supplement: Supplementary file 3 — Authors’ original file for figure 3 [file 12891_2014_2250_MOESM3_ESM.tif]

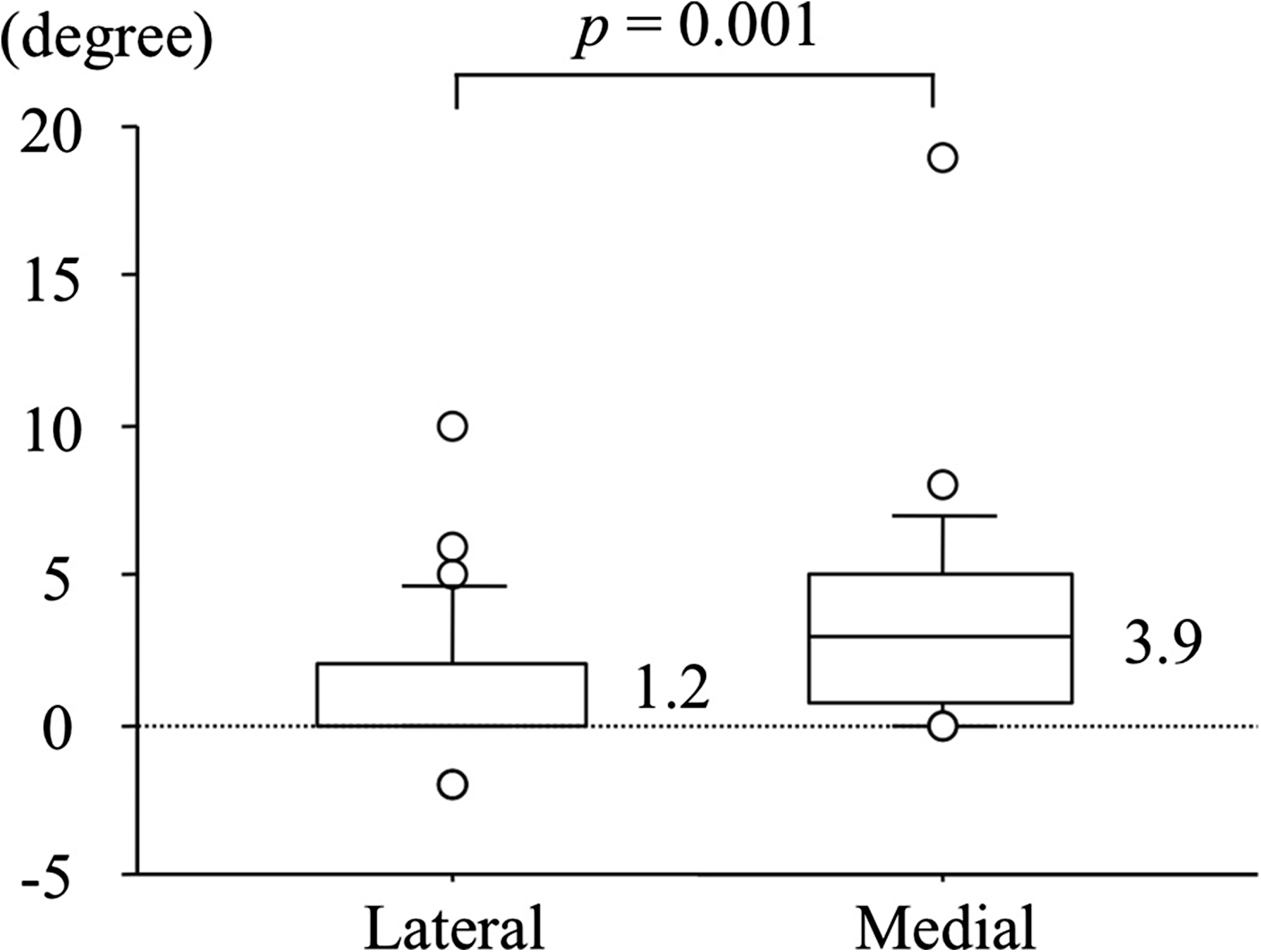

Supplement: Supplementary file 4 — Authors’ original file for figure 4 [file 12891_2014_2250_MOESM4_ESM.tif]
